# Renal impairment is one of appropriate predictors of future diabetic peripheral neuropathy: a hospital-based 6-year follow-up study

**DOI:** 10.1038/s41598-022-09333-3

**Published:** 2022-03-28

**Authors:** Chi-Sheng Wang, Yen-Wei Pai, Ching-Heng Lin, I-Te Lee, Ming-Hong Chang

**Affiliations:** 1 Neurological Institute, Taichung Veterans General Hospital, No. 1650, Taiwan Boulevard, Sec. 4, Taichung City, 40705 Taiwan, ROC; 2 Department of Medical Research, Taichung Veterans General Hospital, No. 1650, Taiwan Boulevard, Sec. 4, Taichung City, 40705 Taiwan, ROC; 3 Division of Endocrinology and Metabolism, Department of Internal Medicine, Taichung Veterans General Hospital, No. 1650, Taiwan Boulevard, Sec. 4, Taichung City, 40705 Taiwan, ROC; 4 Department of Medicine, School of Medicine, Chung Shan Medical University, No.110, Sec. 1, Jianguo N. Road, Taichung City, 40201 Taiwan, ROC; 5 Department of Post-Baccalaureate Medicine, College of Medicine, National Chung Hsing University, No 145, XingDa Road, South Dist., Taichung, Taiwan, ROC

**Keywords:** Endocrinology, Neurology, Risk factors

## Abstract

The relationship between renal impairment and diabetic peripheral neuropathy (DPN) remains inconclusive. We aim to investigate the risk factors for the occurrence of DPN in Taiwanese adults with type 2 diabetes mellitus (T2DM) and focus on renal impairment. A hospital-based study was conducted from 2013 to 2019 and 552 Taiwanese people who had T2DM without DPN at baseline were enrolled. DPN was diagnosed using the Michigan Neuropathy Screening Instrument. Potential risk factors were recorded, including patient’s sociodemographic factors, current medication usage and biochemical markers. As of 2019, 73 developed DPN and 479 had no DPN. The cumulative incidence during the 6-year period was 13.22%. Multivariable logistic regression analysis revealed that lower estimated glomerular filtration rate (eGFR) (odds ratio [OR] 0.98, p = 0.005), advanced age (OR 1.06, p = 0.001), increased body weight (OR 1.04, p = 0.018), duration of DM (OR 1.05, p = 0.036) and male gender (OR 3.69, p = 0.011) were significantly associated with future DPN. In addition, patients with T2DM under the age of 65 with higher serum creatinine concentration (OR 8.91, p = 0.005) and higher baseline HbA1C (OR 1.71, p < 0.001) revealed significantly associated with future DPN. In conclusion, this is the first large scaled hospital-based study with long term follow-up to investigate risk factors for DPN in Taiwanese. Lower eGFR and higher serum creatinine concentration, particularly in people under the age of 65, are predictors of future DPN in Taiwanese people with T2DM. Other predictors included advanced age, increased body weight, duration of DM, male gender for all ages and HbA1c in enrolled patients under the age of 65. Our study not only confirms the association between renal impairment and future DPN but also provides a commonly available assessment to predict the future DPN.

## Introduction

The global burden of diabetes mellitus (DM) has increased enormously in recent decades and will continue to soar in the next few decades. In fact, the global incidence of diabetes has increased by 102.9% from 11.3 million in 1990 to 22.9 million in 2017. Consequently, the prevalence of the complications resulting from type 2 diabetes (T2DM) is likely to rise^1^.

DPN is the most common complication, and its lifetime prevalence is up to 50% in adults with T2DM^2^. DPN is associated with a wide range of clinical manifestations, of which distal sensory neuropathy is predominant. This manifestation contributes to numerous disabling morbidities, such as diabetic foot ulceration, impaired balance, and distressing neuropathic pain, which are often difficult to treat. Furthermore, DPN is the most common cause of non-traumatic lower-limb amputations in most high-income countries^3^. The current study focuses on distal and symmetric polyneuropathy.

Unfortunately, the early manifestations of this insidious disease are often missed until the disease is well established, at which point it seems to be irreversible^2^. There is a lack of treatments that target the underlying nerve damage other than serum glucose control, which shows limited efficacy in T2DM^4^. Thus, prevention is the critical component of diabetes care to reduce the burden of care. Previous studies have reported risk factors that include older age, hyperglycemia, longer diabetes duration, metabolic syndrome and dyslipidemia^5,6^. For dyslipidemia, increased low-density lipoprotein (LDL)^7^ and triglycerides (TG)^8^ have been identified as predictors of diabetic sensory neuropathy in type 1 DM. In contrast, it remained inconclusive in T2DM^10,11^ and there were some studies reported high level of TG and low level of high-density lipoprotein (HDL) as risk factors^3,9^.

Apart from these, it attracts much more attention that whether renal impairment was a predictor of future diabetic peripheral neuropathy. Conflicting data have been reported between renal impairment and future DPN^12,13^. As far as we know, recent studies have not described a definite list of risk factors of DPN, especially renal impairment, which may be due to the majority of studies having cross-sectional designs. Longitudinal studies are the key tools to establish predictors of the development of DPN. Therefore, the objective of the current study was to investigate the predictors for future DPN in Taiwanese adults with T2DM and focus on impaired renal function. Look forward to help improve therapeutic strategies in clinical practice.

## Methods

### Study design and participants

This is a hospital-based, prospective, observational study. Between January 2013 and October 2013, patients over 18 years old with prevalent or newly diagnosed T2DM were eligible for inclusion. The diagnosis of T2DM were based on the criteria of American Diabetes Association (ADA). Data were obtained from patient’s medical records, laboratory examinations, questionnaires and anthropometric measurements at the time of enrollment. Exclusion criteria were as follows: patients having type 1 DM or gestational diabetes, patients had DPN at baseline and whose did not complete the questionnaires or blood sample test at baseline or during the following 6 years. Finally, 552 participants were enrolled in our study.

Participants have been followed observationally via clinical follow-up examination and questionnaires. The blood sample test was performed at least once a year. Our study consequently carried out to 2019—6 years after the trial baseline.

Each of the participants was diagnosed by endocrinologists in the outpatient units at a tertiary medical center in middle Taiwan, which serves approximately 6600 outpatients and 1400 inpatients per day and mainly Han-Chinese population. Before drawn for analysis, the patients’ information was anonymized by computer system, and the researchers were blinded to these data. The study was approved by the Institutional Review Board of Taichung Veterans General Hospital (CG18082B-1). All participants volunteered for the current studies, and provided written informed consent prior to enrolment. Besides, all the methods were performed in accordance with relevant guidelines and regulations.

### Anthropometric measurements

While entry the study, all participants received anthropometric measurements, which was performed by a case-management nurse. The sociodemographic factors included height, weight, waist circumference, duration of diabetes, smoking status and body mass index (BMI). For the details of anthropometric measurements, please refers to our published study^6^. Besides, we recorded the participants’ comorbidities and current medication usage at baseline. Comorbidities obtained from medical record and based on International Classification of Diseases, 9^th^ revision Clinical Modification (ICD-9-CM) and 10^th^ revision (ICD-10) which including hypertension (HTN; ICD-9-CM codes 401–405, ICD-10 codes I10-I15), cerebrovascular disease (CVD; ICD-9-CM codes 430–438, ICD-10 codes I60-I69), ischemic heart disease (IHD; ICD-9-CM codes 410–414, ICD-10 codes I20-I25), liver disease (ICD-9-CM codes 571–573, ICD-10 codes K70-K77). Current medication usage including oral hypoglycemic agent (OHA), insulin, antihypertensive drugs and lipid-lowering drugs such as statins and fibrates.

### Biochemical data

Laboratory examination were administrated during endocrinological follow-up. Blood samples were obtained in the morning after an overnight fasting period from the antecubital vein. Fasting plasma glucose (FPG; using standard enzymatic methods), glycated hemoglobin (HbA1c; using high-performance liquid chromatography), serum creatinine concentration and plasma lipid profiles (using standard enzymatic methods), including total cholesterol (TC), HDL, LDL, and TG. For lipid profile, we defined the following cut-off points of pathologic values according to the National Cholesterol Education Program (NCEP) Adult Treatment Panel (ATP) III^14^: TG: > 150 mg/dL; HDL: < 50 mg/dL in female and < 40 mg/dL in male. EGFR was estimated by the six-variable Modification of Diet in Renal Disease (MDRD) equation as the following equation:

$$186 \times {\text{Serum\,creatinine}}^{-1.154} \times {\text{Age}}^{-0.203} \times (0.742\text{ if\,female})$$

For the patients who had received lipid-lowering drugs, their baseline lipid profiles were defined as the mean plasma lipid values (including TC, HDL, LDL and TG) in the 5 years prior to drug prescription and the follow-up lipid profiles were defined as the mean lipid values in the 5 years after medication prescription. For the individuals never received lipid-lowering drugs, the baseline lipid profiles were circumscribed as the 5-year mean plasma lipid values prior to study enrollment, from 2008 to 2012, and the follow-up lipid profiles were defined as the mean lipid values within the 5 years after study entry, from 2013 to 2017.

### Assessment of diabetic peripheral neuropathy

All of the included patients received assessment of DPN at the time of enrollment and received second assessment after 6-year follow-up by the same trained and certificated care-management nurse to minimize the inter-rater reliability. DPN was evaluated based on the second component of MNSI. Physical appearance of feet, ulceration, ankle deep tendon reflexes, and the perception of light touch (using Semmes–Weinstein 5.07 10-g monofilament) and distal vibration (using 128-Hz tuning fork) were investigated. As previous validated studies in adults^15^, individuals whose MNSI examination (MNSIE) score > 2 were diagnosed with DPN.

### Assessment of renal function

We evaluated participants’ baseline renal function with serum creatinine concentration and eGFR in 2013. The eGFR was estimated by MDRD equation which contains elements as serum creatinine, age and gender (a constant in the equation). Because the relationship between serum creatinine, age and eGFR is hyperbolic, we establish a model that do not adjust the serum creatinine, age and gender for eGFR in multivariable logistic regression analysis (Table 3) to statistic the interference between baseline eGFR and the occurrence of DPN.

Besides, it is well-established that serum creatinine had multiple limitations to represent the true renal function and age is an important factor among these^16^. Furthermore, renal function declines with advancing age. Recent research reported that serum creatinine concentration was not enough to represent a screening test for renal impairment in people aged 65 and above^17^. Because of there were high percentage (14.4% ~ 17% varied by gender) of people aged 65 and above had a serum creatinine concentration above the laboratory reported upper reference limit of normal^18^. Serum creatinine might lead to marked under-investigation and under-recognition of renal failure in this population. Thus we stratified the serum creatinine concentration by age group into age≧65 and age < 65. Each groups were carried out the multivariable logistic regression analysis (Table 4).

In addition, some previous studies revealed medications, baseline glycemic control, and comorbidities might bring about renal impairment^17,19^. Thus we conduct multivariable analysis for renal function which account for these three categories: baseline HbA1c and variables in mediation and comorbidities category that statistical significance level as P value less than 0.1 (P < 0.1) in addition to confounders which had shown a significant correlation. If P value > 0.1, we consider it might play a minor role in incident DPN pathogenesis. Because the relationship between hypertension and antihypertensive drugs is hyperbolic, we choose antihypertensive drugs instead of hypertension. Thus antihypertensive drugs of medication category, cerebrovascular disease of comorbidities category and baseline HbA1c enter the multivariable regression models for baseline eGFR and serum creatinine concentration (Tables 3, 4).

### Statistical methods

Descriptive statistics were presented as the mean values ± standard deviation (SD) and as the numbers with percentages. We used Fisher’s exact test or chi-squared test to analyze categorical variables, while the analyses of continuous variables were conducted using independent t-test or paired t-test.

Multivariable logistic regression analyses were carried out to explore the effect of each identified independent variable on DPN. The multivariable regression models included all the confounders and the variables that had shown a significant correlation, and the adjusted odds ratios (OR) with 95% confidence interval (CI) were calculated between the comparison groups. The statistical significance level chosen was P value less than 0.05 (P < 0.05), and all tests were two-sided. All the data were analyzed using statistical package SAS version 9.4 for Windows.

## Results

We recruited 681 participants who had T2DM at baseline in 2013. Of these, 116 (17%) who had DPN at baseline and 13 non-T2DM patients were excluded. Thus, 552 were deemed to be eligible to be included in the study. The participants’ median age was 59.7 ± 10.7 years, and 60.1% were males. The mean duration of diabetes was 15.2 ± 6.9 years, and the mean level of HbA1c was 7.4 ± 1.3%. Table 1 summarizes their sociodemographic factors, diabetes-related factors, biochemical factors, comorbidities, and medication usage.

**Table 1:** Baseline characteristics of participants in our study.

Variable	Total (n = 552)		Without incident DPN (n = 479)		With incident DPN (n = 73)		*P* value
	N	%	N	%	N	%	
**Sociodemographic factors**							
Age, years	59.7 ± 10.7		58.8 ± 10.4		65.5 ± 10.7		< 0.001
Male gender	332	60.1	272	56.8	60	82.2	< 0.001
Height, cm	163.3 ± 8.3		163 ± 8.3		165.6 ± 8.1		0.014
Weight, kg	68.9 ± 13		68.3 ± 13.2		72.3 ± 11.3		0.016
BMI, kg/m^2^	25.7 ± 4		25.7 ± 4.1		26.4 ± 3.2		0.096
Waist circumference, cm	89.6 ± 10.2		89.5 ± 10.4		90.8 ± 8.7		0.630
SBP, mmHg	130.6 ± 13.1		130.1 ± 13.1		133.5 ± 12.5		0.039
DBP, mmHg	77.8 ± 8.2		77.9 ± 8.1		77.3 ± 8.4		0.558
Smoker	64	11.6	54	11.3	10	13.7	0.547
**Diabetes-related factors**							
FPG, mg/dL	141 ± 39.8		140.3 ± 38		145.2 ± 50.1		0.439
HbA1c, % (mmol/mol)	7.4 ± 1.3		7.3 ± 1.2		7.6 ± 1.7		0.131
Duration of diabetes, years	15.2 ± 6.9		14.9 ± 6.9		17.4 ± 6.9		0.004
Number of OHA used	1.9 ± 1		1.9 ± 1		2.1 ± 1.1		0.245
Number of insulin used	0.3 ± 0.6		0.2 ± 0.6		0.3 ± 0.7		0.218
**Biochemical factors**							
UACR, mg/g	75.7 ± 251.7		72.8 ± 259.1		94.9 ± 196.8		0.440
TG, mg/dL	141.1 ± 138.5		136.7 ± 123.7		169.6 ± 209		0.220
≧150	142	29.6	117	28.2	25	38.5	0.092
HDL-C, mg/dL	52 ± 15.3		52.5 ± 14.9		48.4 ± 16.9		0.048
Female < 50; male < 40	148	31.4	127	31.0	21	33.9	0.647
LDL-C, mg/dL	99.3 ± 31.1		99.8 ± 29.7		95.8 ± 39.4		0.433
TC, mg/dL	166.5 ± 34.6		166.9 ± 32.7		164.1 ± 45.9		0.647
Creatinine, mg/dL	0.9 ± 0.3		0.9 ± 0.3		1.1 ± 0.4		0.001
eGFR, mL/min/1.73m^2^	86.4 ± 26.3		87.7 ± 26.2		77.8 ± 25.1		0.005
GPT, U/L	33.5 ± 25.9		33.4 ± 24.8		34.4 ± 32		0.807
**Comorbidities**							
Hypertension	366	66.3	311	64.9	55	75.3	0.080
Cerebrovascular disease	100	18.1	81	16.9	19	26.0	0.060
Ischemic heart disease	80	14.5	69	14.4	11	15.1	0.881
Liver disease	77	13.9	66	13.8	11	15.1	0.767
**Medication**							
Antihypertensive drugs	341	61.8	289	60.3	52	71.2	0.074
Lipid-lowering drugs	370	67.0	324	67.6	46	63.0	0.433

Data are expressed as mean ± SD for continuous variables and frequency (%) for categorical variables. Differences in continuous variables by independent t-test or paired t-test; differences in categorical variables by Fisher’s exact or chi-squared test.

*BMI* body mass index, *kg* kilograms, *cm* centimeters, *SBP* systolic blood pressure, *DBP* diastolic blood pressure, *FPG* fasting plasma glucose, *HbA1c* glycated hemoglobin, *OHA* oral hypoglycemic agent, *UACR* urine albumin-creatinine ratio, *TG* triglyceride, *HDL-C* high-density lipoprotein cholesterol, *LDL-C* low-density lipoprotein cholesterol, *TC* total cholesterol, *eGFR* estimated glomerular filtration rate, *GPT* alanine aminotransferase.

We defined the patients who developed DPN during follow-up as the “incident DPN” group (n = 73). The cumulative incidence of DPN during 6 years of follow-up was 13.22%. The sociodemographic factors revealed that body weight (72.3 ± 11.3 kg vs. 68.3 ± 13.2 kg, p < 0.05), height (165 ± 8.1 cm vs. 163 ± 8.3 cm, p < 0.05) and the measures of SBP (133.5 ± 12.5 mmHg vs. 130.1 ± 13.1 mmHg, p < 0.05) was significantly higher at baseline in patients with incident DPN than in those without incident DPN. Incident DPN were older (65.5 ± 10.7 years vs. 58.8 ± 10.4 years, p < 0.001) and included more males (82.2% vs. 56.8%, p < 0.001). The diabetes-related factors revealed duration of DM was significantly longer in the incident DPN group (17.4 ± 6.9 years vs. 14.9 ± 6.9 years, p < 0.01).

The biochemical factors revealed serum creatinine concentration (1.1 ± 0.4 mg/dl vs. 0.9 ± 0.3 mg/dl, p < 0.01) were significantly higher at baseline in patients with incident DPN than in those without incident DPN. On the other hand, measures of baseline eGFR (77.8 ± 25.1 mL/min/1.73m^2^ vs. 87.7 ± 26.2 mL/min/1.73m^2^, p < 0.01) and HDL (48.4 ± 16.9 mg/dl vs. 52.5 ± 14.9 mg/dl, p < 0.05) were significantly lower for participants with incident DPN.

Patients’ comorbidities at baseline revealed no significant differences between groups, but HTN (75.3% vs. 64.9%, p = 0.08) and CVD (26.0%% vs. 16.9%, p = 0.06) were more common at baseline in patients with incident DPN than in those without it. The DPN and non-DPN groups showed no significant differences in BMI, waist circumference, smoking status, fasting glucose levels, HbA1c levels, OHA and insulin usage, prescriptions of antihypertensive drugs and lipid-lowering drugs, DBP, urine albumin-creatinine ratio (UACR), pathologic high level of TG and LDL, cholesterol nor alanine aminotransferase levels.

### Multivariable logistic regression model

Table 2 shows the adjusted odds ratio for risk factors of incident DPN from the multivariable logistic regression model. Advanced age was associated with an increased risk of DPN (odds ratio [OR] 1.06 [95% CI 1.02; 1.09], p = 0.001). Increased weight (OR 1.04 [95% CI 1.01; 1.07], p = 0.018) and male gender (OR 3.69 [95% CI 1.35; 10.09], p = 0.011) were significantly associated with a higher risk of DPN. Duration of DM (OR 1.05 [95% CI 1.00; 1.09], p = 0.036) was significantly associated with a higher risk of DPN as well. In contrast, height, lower HDL and baseline SBP revealed no statistically significant associations with the risk of DPN after adjustment for all confounding factors.

**Table 2:** Risk factors of future DPN in multivariable logistic regression.

Variable	Adjusted OR^a^	95% CI	p-value
**Sociodemographic factors**			
Age, years	1.06	1.02–1.09	0.001
Gender, male	3.69	1.35–10.09	0.011
Height, cm	0.99	0.93–1.06	0.835
Weight, kg	1.04	1.01–1.07	0.018
SBP, mmHg	1.01	0.99–1.03	0.449
**Diabetes-related factors**			
Duration of diabetes, years	1.05	1.00–1.09	0.036
**Biochemical factors**			
eGFR, mL/min/1.73m^2^	0.99	0.98–1.00	0.180
HDL-C, mg/dL	1.00	0.98–1.02	0.910

*OR* odds ratio, *CI* confidence interval, *SBP* systolic blood pressure, *eGFR* estimated glomerular filtration rate, *HDL-C* high-density lipoprotein cholesterol.

^a^Multivariable logistic regression was adjusted for all variables in Table 2.

### Comparison of baseline renal function between patients with or without incident DPN

After adjusted for height, weight, SBP, duration of diabetes, HbA1c, eGFR, HDL-C, cerebrovascular disease and antihypertensive drugs, we found that higher baseline eGFR (OR 0.98 [95% CI 0.97; 0.99], p = 0.005) was significantly associated with a lower risk of DPN (Table 3). In addition, the stratified analysis also revealed that a higher baseline serum creatinine concentration (OR 8.91 [95% CI 1.92; 41.27], p = 0.005) was independently and significantly associated with incident DPN in enrolled patients under the age of 65 (Table 4). In contrast, there were no significant associations between baseline serum creatinine concentration (OR 0.77 [95% CI 0.17; 3.40] p = 0.728) and incident DPN in the elderly (age≧65 years, Table 4). Besides, baseline HbA1c revealed significantly associated with incident DPN (OR 1.71 [95% CI 1.30; 2.24], p < 0.001) in people under the age of 65 in Table 4.

**Table 3:** Odds ratios and 95% confidence intervals for the relationship between eGFR and future DPN.

Variable	Adjusted OR^a^	95% CI	p-value
**Sociodemographic factors**			
Height, cm	1.04	0.99–1.09	0.155
Weight, kg	1.03	1.00–1.06	0.071
SBP, mmHg	1.02	1.00–1.04	0.106
**Diabetes-related factors**			
Duration of diabetes, years	1.06	1.02–1.11	0.006
HbA1c, % (mmol/mol)	1.22	1.00–1.49	0.050
**Biochemical factors**			
eGFR, mL/min/1.73m^2^	0.98	0.97–0.99	0.005
HDL-C, mg/dL	0.99	0.97–1.01	0.433
**Comorbidities**			
Cerebrovascular disease	1.17	0.59–2.35	0.652
**Medication**			
Antihypertensive drugs	0.89	0.45–1.77	0.743

*OR* odds ratio, *CI* confidence interval, *SBP* systolic blood pressure, *HbA1c* glycated hemoglobin, *eGFR* estimated glomerular filtration rate, *HDL-C* high-density lipoprotein cholesterol.

^a^Adjusted for height, weight, SBP, duration of diabetes, HbA1c, eGFR, HDL-C, cerebrovascular disease and antihypertensive drugs.

**Table 4:** The association between serum creatinine concentration and future DPN, stratified by age group into age < 65 and ≧ 65.

Variable	Adjusted OR^a^	95% CI	p-value
**Age < 65**			
Sociodemographic factors			
Age, years	1.09	1.01–1.18	0.037
Gender, male	3.74	0.78–18.01	0.100
Height, cm	0.93	0.84–1.03	0.160
Weight, kg	1.06	0.01–1.12	0.013
SBP, mmHg	1.03	1.00–1.07	0.070
Diabetes-related factors			
Duration of diabetes, years	1.05	0.99–1.13	0.105
HbA1c, % (mmol/mol)	1.71	1.30–2.24	< 0.001
Biochemical factors			
Creatinine, mg/dL	8.91	1.92–41.27	0.005
HDL-C, mg/dL	0.99	0.95–1.02	0.520
Comorbidities			
Cerebrovascular disease	0.72	0.23–2.28	0.577
Medication			
Antihypertensive drugs	0.36	0.12–1.04	0.060
**Age ≧ 65**			
Sociodemographic factors			
Age, years	1.06	0.98–1.13	0.138
Gender, male	2.69	0.65–11.15	0.172
Height, cm	1.04	0.94–1.14	0.456
Weight, kg	1.04	0.99–1.10	0.128
SBP, mmHg	0.99	0.95–1.03	0.650
Diabetes-related factors			
Duration of diabetes, years	1.03	0.96–1.11	0.369
HbA1c, % (mmol/mol)	0.81	0.48–1.37	0.438
Biochemical factors			
Creatinine, mg/dL	0.77	0.17–3.40	0.728
HDL-C, mg/dL	1.00	0.97–1.04	0.861
Comorbidities			
Cerebrovascular disease	1.16	0.42–3.17	0.773
Medication			
Antihypertensive drugs	1.73	0.49–6.04	0.392

*OR* odds ratio, *CI* confidence interval, *SBP* systolic blood pressure, *HbA1c* glycated hemoglobin, *HDL-C* high-density lipoprotein cholesterol.

^a^Each group was adjusted for age, gender, height, weight, SBP, duration of diabetes, HbA1c, creatinine, HDL-C, cerebrovascular disease and antihypertensive drugs.

## Discussion

To our knowledge, this is the first large scaled observational study to investigate risk factors for DPN in a Taiwanese adult population. Using MNSIE for the diagnosis of DPN, we found that participants without DPN at baseline had a 13% cumulative incidence of DPN over the 6 years of follow-up (corresponding with an annual incidence of 2.204%) in a population where the duration of DM was as long as 15.2 ± 6.9 years. The incidence of DPN in our study is comparable with that of a previous longitudinal, large-scale, nationwide, population-based study in Taiwan (n = 37,375, annual incidence of 3.2%)^20^ However, it was lower than that reported Western populations^21,22^. This discrepancy might be due to differences in the sample size, ethnicity of the study population (the prevalence of DPN is about 32.1% in the UK^23^ and about 23.5% in Taiwan^24^), diagnostic criteria, and measurement instruments.

Apart from these, one of the crucial factors is the baseline duration of DM. One study well established that the prevalence of diabetic neuropathy increased from 8 to 42% in patients with T2DM when patients were monitored for 10 years^25^. Compared with the previous longitudinal study, patients had newly diagnosed DM with a cumulative incidence of 10% over the 13-year follow-up period and an annual incidence of 0.7%^9^. The relatively high cumulative incidence over our 6-year follow-up period might be attributable to the longer baseline duration of DM.

### The association between renal function and incident DPN

In our study, baseline renal function was found to be an independent risk factor for DPN, including baseline eGFR (Table 3) and baseline serum creatinine concentration (Table 4), particularly in patients under the age of 65. This finding was inconsistent in patients aged 65 and above, which might be due to the decline of renal function in the aging process. This is consistent with the Rochester cohort longitudinal assessment^12^, in which Dyck et al. reported that the presence of DPN is associated with the severity of nephropathy and might be implicated in its cause. Our previous studies also indicate that the prevalence of DPN increases significantly in patients with impaired renal function^6^. On the other hand, the baseline UACR did not show the same result, which may be attributed to the large standard deviations (94.9 ± 196.8 mg/g vs. 72.8 ± 259.1 mg/g, p = 0.44).

To date, the mechanisms of neurotoxicity in T2DM patients with renal impairment remains unclear, but they have been demonstrated in some studies^26,27^. Experimental evidence indicates that renal impairment result in alteration in membrane excitability which is induced by inhibition of the axonal Na^+^/K^+^ pump. Consequently, it abolishes the direct contribution of the hyperpolarizing pump current to the membrane potential, leading to an accumulation of extracellular K^+^ that causes depolarization^28^. Disruption of these various ionic gradients may affect the Na^+^/Ca^2+^ exchanger, leading to increased levels of intracellular Ca^2+^ and axonal loss^29^.

In addition, it is clear from previous research that impaired renal function results in microvascular endothelial dysfunction, even in the early stages of chronic kidney disease. Endothelial injury is caused by various factors, including inflammation, hypertension, diabetes-associated factors, and a uremic milieu^27,30^. Eventually, it leads to neuropathy due to impaired nerve blood flow, epineurial arterio-venous shunting, and reduced nerve oxygen tension^31^.

Other studies examining nephropathy as a risk factor for DPN have been inconclusive^13^. However, it is suggested that the selection of disease markers for renal impairment may be important (for example, eGFR or creatinine), and further investigation is needed. Based on the current study, we recommend that increased serum creatinine concentration or lower baseline eGFR be used as an indicator to enhance the awareness of incident DPN.

### Other risk factors of future DPN

After adjustment for potential confounding factors, we also found that a higher risk of DPN was linked with increased age, body weight, duration of DM, and male gender. Our findings are consistent with most previous reports from cross-sectional studies and a meta-analysis of patients with T2DM in Western, Korean, and Taiwanese populations^5,6,32^. Concerning sugar control, previous studies indicated hyperglycemia as a risk factor for the development of DPN^5,8^, but we found no association between baseline HbA1c levels and incident DPN. This is likely explained by low levels of HbA1c at baseline (7.3 ± 1.2% in the no-DPN group and 7.6 ± 1.7% in the incident-DPN group) compared with the levels usually found in previous studies. These data possibly reflect better medication adherence among Taiwanese DM patients^33^ compared with worldwide^34^. Our study also showed equally high numbers of hypoglycemic medication prescriptions in both groups. On the other hand, baseline HbA1c was found to be an independent risk factor for DPN in enrolled patients under the age of 65 (Table 4) but not in all ages. This finding might be attributed to the effect of age on the HbA1c. A possible explanation is that elderly individuals encounter physiologically decreased RBC count thus HbA1c is unsuitable for a marker of glycemic control in elderly^35^. In summary, the result implies us that baseline glycemic control might play a role in incident DPN pathogenesis in people under the age of 65 but further research is warranted.

In the current study, increased weight was independent risk factor of incident DPN, but no statistically significant associations with incident DPN were found for BMI and waist circumference. This is inconsistent with previous studies^5,9,10^ but previous studies have not identified a consistent list of risk factors related to markers of obesity^10,12^. A possible explanation is that previous investigators did not adequately correct the reference cut-off values and the units for tests. This is not to say that markers of obesity may not be risk factors for DPN, but corrections must first be made for these characteristics in the cut-off values and the units^12^.

In terms of dyslipidemia, we found that serum lipid components had no statistically significant associations with the risk of DPN in T2DM. As stated above, these findings were consisted with some previous studies^36,37^. In fact, accumulated evidence has shown a correlation between DPN and serum lipid profiles but has shown inconsistent results^38^. The possible underlying mechanisms of dyslipidemia leading to DPN are complex which may include insulin resistance, chronic inflammatory status, oxidative stress induced by elevated LDL, and demyelination^38^. Nevertheless, these mechanisms are mainly reported in preclinical studies^39–41^. It is well established that DPN is a multifactorial disease and our findings indicate that lipid metabolism may play a minor role in its pathogenesis.

The major strengths of the current study are its large sample size with long term follow-up, the unselected nature of participants, standardized data collection procedures, and inclusion of several potential risk factors at baseline. But despite these strengths, there are still plenty of limitations. First, our results might not apply to treatment-naïve cohorts of early-stage T2DM. A high proportion of medication prescription might have affected the cardiovascular risk factors. Furthermore, we did not use confirmatory tests such as nerve conduction studies or skin biopsy for DPN diagnosis. However, the diagnosis of DPN is principally a clinical one according to ADA recommendations, and the MNSIE is a sensitive, specific, validated clinical screening tool. Lastly, we included participants from a single hospital, which might limit the generalizability of the results.

## Conclusion

Lower eGFR and higher serum creatinine concentration, particularly in people under the age of 65, are predictors of future DPN in Taiwanese people with T2DM. Other risk factors included advanced age, increased body weight, duration of DM, male gender for all ages and HbA1c in enrolled patients under the age of 65 which were compatible with most previous studies. These findings not only confirm the association between renal impairment and future DPN but also provides a commonly available assessment to predict the future DPN. Early detection of risk factors and control of the modifiable factors could enrich therapeutic strategies in clinical practice. Thus, we suggest that the therapeutic strategy for diabetes should provide early management of impaired renal function and prevent overweight. Also, these findings could provide useful information for researchers exploring the underlying mechanisms of DPN and inspire disease-modifying therapies in the future.

## Data Availability

The datasets generated during and/or analyzed during the current study are available from the corresponding author on reasonable request.
